# Evaluation of Ergonomics-Related Disorders in Online Education Using Fuzzy AHP

**DOI:** 10.1155/2021/2214971

**Published:** 2021-09-27

**Authors:** Hemant Upadhyay, Sapna Juneja, Abhinav Juneja, Gaurav Dhiman, Sandeep Kautish

**Affiliations:** ^1^BMIET, Sonepat, India; ^2^IMS Engineering College, Ghaziabad, India; ^3^KIET Group of Institutions, Delhi NCR, Ghaziabad, India; ^4^Government Bikram College for Commerce, Patiala, India; ^5^LBEF Campus, Kathmandu, Nepal

## Abstract

The aim of the presented work is to analyze the ergonomics-related disorders in online education using the fuzzy AHP approach. A group dialogue with online education academicians, online education students, biotechnologists, and sedentary computer users has been performed to spot ergonomics-related disorders in online education. Totally eight ergonomics-related disorders in online education have been identified, and the weight of each disorder has been computed with triangle-shaped fuzzy numbers in pairwise comparison. Furthermore, the ergonomics-related disorders in online education are kept in four major categories such as afflictive disorders, specific disorders, psychosocial disorders, and chronic disorders. These four categories of ergonomics-related disorders in online education are evaluated and compared using fuzzy analytical hierarchical process methodology to get ranked in terms of priorities. The results may be instrumental for taking appropriate corrective actions to prevent ergonomics-related disorders.

## 1. Introduction

The international associations have termed ergonomics to be “the design of work, in such a manner that human competencies can be utilized in the best possible manner without overcoming human constraints” [1]. Ergonomics is the scientific know-how of the man at work, with the numerous psychosocial and medical characteristics of human work. The practical objective of ergonomics is the conditioning and justification of the adaptation of work to man [2]. The ergonomics literature such as that of Hünting et al. [3], Sauter et al. [4], and Berqvist et al. [5] have been mostly on the basis of desktop and laptop computers.

Several documents of reputed research and academic institutions [6] indicate common ergonomics-based documentation. These days, a large number of studies on computer-human interfaces have introduced newer ergonomics criteria and recommendations [2]. It has been observed that if essential precautions were not considered for inappropriate and very frequent computer utilization in our day-to-day lives, a considerable enhancement is noted in the number of disorder practicing persons as a result of the screen time with laptop or desktop for a longer duration [7]. Heiden et al. [8] suggest that by accommodating subjective rankings by the employees as well as objective computation of the work surroundings, an analysis can be made to develop an exhortation regarding light availability and further emphasize the relevance that visual surrounding factors like glare are the reason for headache and eyestrain. The presented paper is an investigation work in ergonomics of computer-human interface that recommends the evaluation of ergonomics-related disorders in online education according to four main categories such as afflictive disorders, special disorders, psychosocial disorders, and chronic disorders and presents the ability of fuzzy AHP methodology in selecting and prioritizing of above criteria.

The presented paper has been set as follows.  Section 2 is about online education and types of ergonomics-related disorders in online education. Fuzzy AHP methodology is described in Section 3. A proposed hierarchical model for categorization of ergonomics-related disorders has also been discussed in Section 3.  Section 4 brings the results. Finally, in Section 5, conclusions are drawn, and future scope is mentioned.

## 2. Literature Review

People who have been permanent stakeholders of the information world can explore a virtual-based life available in the World Wide Web at the computer system for data searching. Various in-service learning events are conducted for making the teachers be well-versed with computer engineering and information and communication technologies for playing a significant role in the execution of these activities [9]. A variety of commercial companies are linked with the growth of the progression of e-learning [10]. A teacher facilitates a knowledge analyst by demonstrating the subject's concept [11].

**Figure 1:**
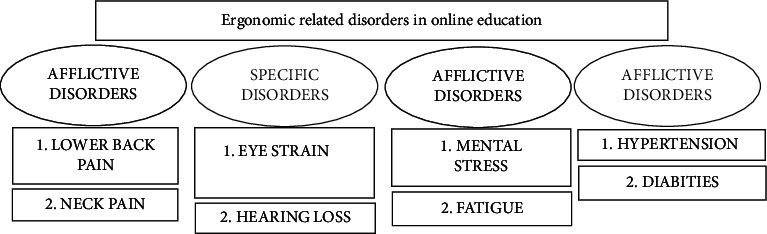
The proposed fuzzy AHP-based hierarchical model.

The epidemic caused by lockdown has enhanced the use of the online mode of learning and forced academicians to adjust education mode [12]. It is notable that online education surroundings are developed and analyzed in academic patterns by considering both utility and erudition aspects [13]. The unparallel magnification of web-based engineering is leading towards the emergence of several methods for the area of academic visibility in the online education scenario [14]. The objective for the utilization of online education technology is to modify the way of teaching in academic institutions from the conventional approach towards the more participated and interactive [15]. The recent changes in online learning have witnessed unprecedented growth in the last couple of years; furthermore, the current pandemic condition has accelerated the process of online learning management [16]. Recently, the pandemic has changed all aspects of our lives. Social isolation has deranged conventional educational practices and has influenced traditional schooling and training. There is an intense requirement to innovate and implement alternative educational and evaluation procedures. Next, ergonomics-based adverse effects related to sedentary sitting have been shortlisted in different categories. Fuzzy analytical hierarchical process-based hierarchical model of eight chosen ergonomics-related disorders in four categories has been shown in Figure 1.

### 2.1. Afflictive Disorders

**Figure 2:**
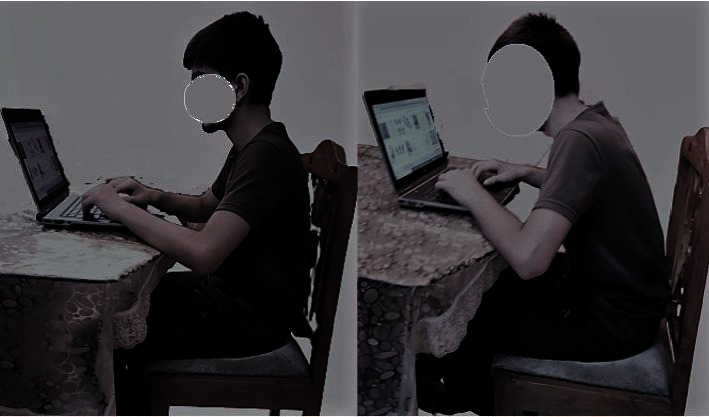
Poor working posture impacting lower back pain and neck pain.

Afflictive disorders are a set of circumstances affecting the joints, tendons, and muscles [17]. These can impact the lower back, shoulders, neck, and wrists. These are usually because of a poor working posture [18]. The afflictive disorders can be visualized in Figure 2 as how poor working posture with sedentary sitting can impact the lower back and neck.

#### 2.1.1. Lower Back Pain

The application of computers and laptops and sedentary sitting may develop ascendancy on the lower part of the back of desktop users, as a result of inadequate posture [19]. Low back pain is practiced by computer users who have to work with a keyboard while in a sedentary position [20]. Lower back pain simultaneously creates effects on other muscles resulting in pain [21].

#### 2.1.2. Neck Pain

There is a strong relation between sedentary sitting and pain in the neck, with an affirmative association of pain in neck and neck flexion [22]. There is a larger decline to be linked with less menace of musculoskeletal strain in the neck [23].

### 2.2. Specific Disorders

#### 2.2.1. Eyestrain

Eyestrain is a family of vision or eyesight-related issues that result from a longer use of computer system, tablet, or mobile phone [24]. Eye discomfort and visual problems have been linked to computerized tasks [25]. The abundance of CVS in computer system regular users is indeed linked to the screen time duration of the computer system [26].

#### 2.2.2. Hearing Loss

Enhancing measures of unprecedented occupational and surrounding commotion have become the prime reasons for commotion-generated hearing loss and form a vital public health issue [27]. The application of individual instruments for listening has been recognized as a prime cause in the expansion of noise-induced ear issues [28]. The stereotypically used devices are the earbud fashioned headphones that have been related to a highly demanded and absolute measure of listening [29].

### 2.3. Psychosocial Disorders

The management of psychosocial factors is often not psychosocial in nature. At times, it may be very tedious to correct psychosocial conditions directly [30].

#### 2.3.1. Mental Stress

The definition of “stress” may be a general reaction to a stressor, consisting of several physiological reactions. Many modeling frameworks demonstrate that detrimental psychosocial issues become the reason behind mental stress [23]. Several studies emphasize the importance of stress [31]. The longer the sedentary period, the higher the perceived stress score; it is significant up to a great extent [32].

#### 2.3.2. Fatigue

To spot the manifestation of ergonomic disorders, a questionnaire [33] was used enabling recognition of parts of the body where individuals practiced symptoms of fatigue. It is unfounded that interventions that focus on sitting less and moving more often increase fatigue [34].

### 2.4. Chronic Disorders

#### 2.4.1. Hypertension

Numerous studies have demonstrated correlations between significant Internet use and health issues such as hypertension. Screen time has been related to enhanced blood pressure independent of body composition (American Heart Association). Interrupting sedentary sitting on the computer may considerably decrease hypertension [35].

#### 2.4.2. Diabetes

Statements of internationally recognized agencies show that diabetes has enclosed particular recommendations to decrease sedentary sitting of computer users [36]. Continuous and everyday stable sitting hours on computers may be related to poorer health outcomes in those with diabetes. Dempsey et al. [37] demonstrated a relation between diabetes and a day of sedentary sitting on a computer.

## 3. Methodology

### 3.1. Fuzzy AHP

The current work has proposed the use of fuzzy analytical hierarchical process methodology in the analysis of ergonomics-related disorders in online education. The AHP, firstly discussed by Saaty [38], proposed a method for the calculation of the relative importance in an MCDM problem [39]. The traditional analytical hierarchical process is not sufficient to manage the ambiguous characteristic of lingual analysis [40]. In analytical hierarchical process, all comparisons are not included [41]. Analytical hierarchical process is unable to deal with its constraints such as (a) analytical hierarchical process generates the decisions in crisp nature and has been considered as an approach with less accuracy and (b) assessment of alternatives, done by the group of experts, is based on approximation and is the intuitive analysis of approximate computation [42]. The utilization of fuzzy set theory [43] may permit the decision-makers to accommodate qualitative data, improper data, and ignorant facts in the decision model [44]. The evolution of the fuzzy analytical hierarchical process has shown its capabilities for the resolution of complex problems with considerable accuracy [45]. The fuzzy analytical hierarchical process model is very instrumental in the ranking of nonobjective factors [46]. Even after complex calculations, fuzzy AHP is able to deal with the general appraisal of ambiguity because of human tendency [47]. The advantage of fuzzy logic is much accurate computation of the factors prioritizing and hierarchy creation [48]. Popular technique, fuzzified analytical hierarchical process is capable of solving the complicated problems related to selection of vendors [49]. The utilization of the fuzzy analytical hierarchical process has been becoming much popular in several specializations due to its ability to work as disintegration technique [50].

The aim of F-AHP is to cope with complicated decision science issues using a hierarchy with main criteria and the computation of eigenvectors [51]. Fuzzy AHP is instrumental in assessing pairwise comparative study, alternatives, and factors [52]. Triangle fuzzy numbering structures are utilized for pairwise comparative tables [53]. The geometric mean technique assists in computing the fuzzy weightages and hierarchic rank system of the factors [54]. Numerous mathematical methods are available for the deriving process from fuzzy pairwise comparative matrices into crisp weights [55]. Fuzzy AHP is a simple and presentiment technique with appropriate validation of consistent index [56]. Past relevant work exploration shows that fuzzy analytical hierarchical process has the potential to get several complex multicriteria decision-making problems solved as some research works are mentioned in Table 1.

### 3.2. Proposed Model

**Figure 3:**
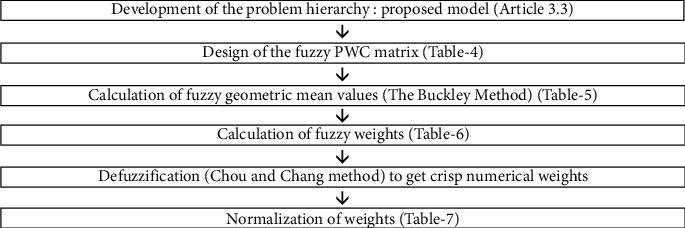
The research framework deployed in this study.

Fuzzy AHP is a modified version of an analytic hierarchy process with the theory based on fuzzified logic. Fuzzy analytic hierarchy process frames the fuzzy triangular scale consisting of lower, middle, and upper values for computation of priorities. We have three frequently employed fuzzy analytical hierarchical process methods but probably the first fuzzy AHP technique presented by van Laarhoven and Pedrycz [60]. Mikhailov [61] suggested a fuzzy programming process to determine optimized crisp priorities that are achieved with fuzzy PWC judgments. Chang [62] suggested a unique method of integers and fractions in triangular structure in pairwise CMs. Afterwards, Buckley [63] extended the study by computing the fuzzy priority of each comparative ratio. Pairwise comparative matrices are allocated to elements of the analysis hierarchy of the Saaty scale [64, 65]. Buckley's technique [63] is instrumental in computing the relative weightages for solutions as well as factors in fuzzy AHP. This methodology suggests the utilization of geometric means achieving fuzzy weightages that enhance the ease for computation of the local weightages [66]. The research framework of the present study has been shown in Figure 3.

We have different types of fuzzy numbers, but the triangle shape fuzzy numbers have been mostly applied, and the number structure is a set of three numbers (lower, medium, and upper). Real number values (lower, medium, and upper) have the triangle structure as “*l*,” “*m*,” and “*n*” as the least probability number, the most probability value, and the largest probability number, respectively [21]. Linguistic-based scaling for triangle fuzzy numbers has been indicated in Table 2.

### 3.3. Proposed Hierarchy

Abbreviations of the chosen ergonomics-related disorders have been category-wise proposed in Table 3.

## 4. Results

For the validation of our proposed model, a group dialogue with online education academicians, online education students, biotechnologists, and sedentary computer users has been performed in detail. We demonstrate the formation of fuzzy pairwise comparison of ergonomics-related disorders in online education (Table 4).

Furthermore, the GM of fuzzy compared values of each ergonomics-related disorder in online education is calculated as shown in Table 5.

Furthermore, the fuzzy weight of every ergonomics-related disorder in online education is computed in Table 6. Lower, medium, and upper fuzzy weights have been calculated for each ergonomics-related disorder by multiplying respective geometric means with the specified factor (as calculated in the last row of Table 5).

Furthermore, the crisp values of the weight of every ergonomics-related disorder (Mi) have been computed as the arithmetic mean of fuzzified values for every disorder. Finally, the crisp weights of every ergonomics-related disorder are put after normalization as Ni as tabulated in Table 7.

Table 7 shows that neck pain (D2) has the least-normalized relative weight among the chosen ergonomics-related disorders in online education.

**Figure 4:**
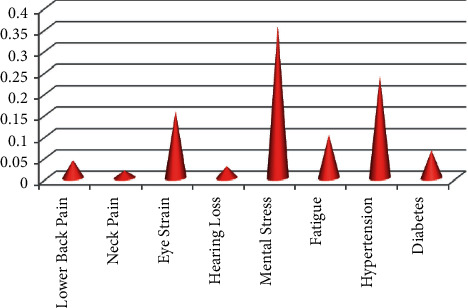
Normalized relative weights of ergonomics-related disorders.

It has been shown in Figure 4 that mental stress has the greatest normalized relative weight among the discussed ones. Hypertension has the second-highest normal weightages, and eyestrain has the third-highest normal weightages. On the other hand, neck pain has the least normal weight out of all eight considered ergonomics-related disorders in online education.

Table 8 depicts the priority computation by fuzzy analytical hierarchical process method and the rank calculation of the different subcriteria. It has been found that mental stress is the most important, followed by hypertension and eyestrain as an ergonomics-related disorder in online education.

**Figure 5:**
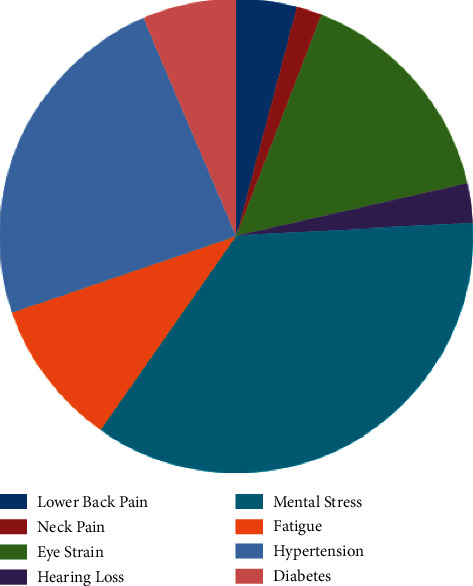
Pie chart of priorities of ergonomics-related disorders.

It has been shown in Figure 5 that priority of “neck pain” is the least in selected ergonomics-related disorders. Figure 5 is the demonstration of priorities of all eight considered ergonomics-related disorders with the help of a pie chart. Different colors are presenting priorities of different ergonomics-related disorders.

Table 9 expresses the priority computed using the fuzzy analytical hierarchical process method.

Based on the values of Table 9, psychosocial disorders have the first place, followed by chronic disorders, specific disorders, and then afflictive disorders.

**Figure 6:**
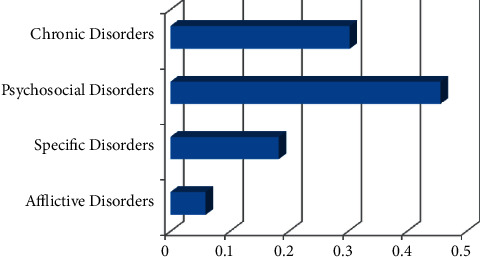
Priority of categories of ergonomics-related disorders.

Figure 6 signifies that the weightage of “psychosocial disorders” is the most (45.7%), and this is the most important ergonomics-related disorder category among the discussed ones.

**Figure 7:**
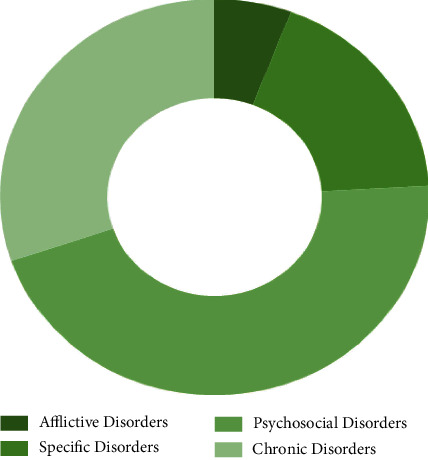
Weightage of categories of ergonomics-related disorders.

It has been shown in Figure 7 that the priority of “afflictive disorders” category is the least (5.8%) ranked ergonomics-related disorder category in the chosen ones.

The outcome of the work by some of the similar efforts has been summarized in Table 10.

The prime objective of the new specialization of “human factors and ergonomics” is its exploration for applying of much ultramodern design structures for current era workplaces. Engineering will witness the evolution of the novel “human factors and ergonomics” domain in upcoming decades [70].

Upcoming developments will emphasize on the aligning of human-machine interface and ergonomics areas for sustainable progress, user safety, and psychophysical issues [71].

## 5. Conclusion

This research work has demonstrated an MCDM modeling for the evaluation of ergonomics disorders analysis in online education applying the fuzzy AHP technique. Eight ergonomics-related disorders in online education were recognized as lower back pain, neck pain, eyestrain, hearing loss, mental stress, fatigue, hypertension, and diabetes. Mental stress has emerged as the most influential ergonomics-related disorder in online education, followed by hypertension and eyestrain. These ergonomics-related disorders in four categories (afflictive disorders, specific disorders, psychosocial disorders, and chronic disorders) have been compared and ranked. Based on calculated mathematical values, “psychosocial disorders” have been ranked the first position followed by “chronic disorders” and “specific disorders.”

The results may be instrumental for taking appropriate corrective actions to prevent ergonomics-related disorders. For numerous industries and corporate organizations, the discussed analysis can play a vital role to enhance the productivity of manpower especially related to sedentary computer screen work for long hours.

This study could consider ergonomics-related disorders in online education with only one methodology. The prospect of further work can be the development of a similar model by applying other multiple criteria decision-making methods available such as best-worst method and so on. Modern MCDM techniques such as case-based reasoning and data envelopment analysis may be used for much-detailed problems in this domain.

## Figures and Tables

**Table 1:** Recent works on applications of fuzzy AHP.

S. No.	Author	Objective and outcome
1	Akbar et al.	The authors [57] prioritized the factors about the scaling procedure of agile methodology for the GSD industry of a particular nation and developed their taxonomy using fuzzy analytical hierarchical process approach.
2	Sunday Oyinlola Ogundoy and Ismaila Adeniyi Kamil	The authors [58] used the fuzzy AHP to rank trust factors in fog computing. They observed the most- and least-preferred factors. Moreover, they considered the subcategory of every criterion for ranking them using the fuzzy analytical hierarchical process approach.
3	Kumar et al.	The authors [59] developed the preference-based categorization of 21 software processing development success attributes using the fuzzified AHP.

**Table 2:** Satty-scale-based linguistic-based scaling for triangle fuzzy numerals.

Scale	Lingual scale for relative weightage	Fuzzified values	Respective values
1	Identical superiority	(1, 1, 1)	(1, 1, 1)
2	Identical to weak superiority	(1, 2, 3)	(1/3, 1/2, 1)
3	Weak superiority	(2, 3, 4)	(1/4, 1/3, 1/2)
4	Weak to moderate superiority	(3, 4, 5)	(1/5, 1/4, 1/3)
5	Moderate superiority	(4, 5, 6)	(1/6, 1/5, 1/4)
6	Moderate to strong superiority	(5, 6, 7)	(1/7, 1/6, 1/5)
7	Strong superiority	(6, 7, 8)	(1/8, 1/7, 1/6)
8	Very strong superiority	(7, 8, 9)	(1/9, 1/8, 1/7)
9	Extreme superiority	(9, 9, 9)	(1/9, 1/9, 1/9)

**Table 3:** The abbreviations of ergonomics-related disorders.

Criteria	Disorder	Abbreviation
Afflictive disorders	Lower back pain	Di1
	Neck pain	Di2

Specific disorders	Eyestrain	Di3
	Hearing loss	Di4

Psychosocial disorders	Mental strain	Di5
	Fatigue	Di6

Chronic disorders	Hypertension	Di7
	Diabetes	Di8

**Table 4:** Fuzzy pairwise comparison of ergonomics-related disorders in online education.

Disorder	Di1	Di2	Di3	Di4	Di5	Di6	Di7	Di8
Di1	(1, 1, 1)	(3, 4, 5)	(1/6, 1/5, 1/4)	(2, 3, 4)	(1/8, 1/7, 1/6)	(1/5, 1/4, 1/3)	(1/7, 1/6, 1/5)	(1/4, 1/3, 1/2)
Di2	(1/5, 1/4, 1/3)	(1, 1, 1)	(1/8, 1/7, 1/6)	(1/4, 1/3, 1/2)	(1/9, 1/9, 1/9)	(1/7, 1/6, 1/5)	(1/9, 1/8, 1/7)	(1/6, 1/5, 1/4)
Di3	(4, 5, 6)	(6, 7, 8)	(1, 1, 1)	(5, 6, 7)	(1/5, 1/4, 1/3)	(2, 3, 4)	(1/4, 1/3, 1/2)	(3, 4, 5)
Di4	(1/4, 1/3, 1/2)	(2, 3, 4)	(1/7, 1/6, 1/5)	(1, 1, 1)	(1/9, 1/8, 1/7)	(1/6, 1/5, 1/4)	(1/8, 1/7, 1/6)	(1/5, 1/4, 1/3)
Di5	(6, 7, 8)	(9, 9, 9)	(3, 4, 5)	(7, 8, 9)	(1, 1, 1)	(4, 5, 6)	(2, 3, 4)	(5, 6, 7)
Di6	(3, 4, 5)	(5, 6, 7)	(1/4, 1/3, 1/2)	(4, 5, 6)	(1/6, 1/5, 1/4)	(1, 1, 1)	(1/5, 1/4, 1/3)	(2, 3, 4)
Di7	(5, 6, 7)	(7, 8, 9)	(2, 3, 4)	(6, 7, 8)	(1/4, 1/3, 1/2)	(3, 4, 5)	(1, 1, 1)	(4, 5, 6)
Di8	(2, 3, 4)	(4, 5, 6)	(1/5, 1/4, 1/3)	(3, 4, 5)	(1/7, 1/6, 1/5)	(1/4, 1/3, 1/2)	(1/6, 1/5, 1/4)	(1, 1, 1)

**Table 5:** Computation of geometric means of different disorders.

Disorders	(Lower, medium, and upper) geometric means		
Di1	(1/1120)^1/8^	(1/210)^1/8^	(1/36)^1/8^
Di2	(1/544320)^1/8^	(1/181440)^1/8^	(1/45360)^1/8^
Di3	(36)^1/8^	(210)^1/8^	(1120)^1/8^
Di4	(1/30240)^1/8^	(1/6720)^1/8^	(1/1260)^1/8^
Di5	(45360)^1/8^	(181440)^1/8^	(544320)^1/8^
Di6	(1)^1/8^	(6)^1/8^	(35)^1/8^
Di7	(1260)^1/8^	(6720)^1/8^	(30240)^1/8^
Di8	(35)^1/8^	(6)^1/8^	(1)^1/8^
Sum	10.293	12.616	15.116
Reciprocal	1/(10.293)	1/(12.616)	1/(15.116)
Increasing order	**1/(15.116)**	**1/(12.616)**	**1/(10.293)**

**Table 6:** Computation of fuzzified weights.

Disorders	Relative fuzzy weightage		
Di1	(1/15.116) (1/1120)^1/8^	(1/12.616) (1/210)^1/8^	(1/10.293) (1/36)^1/8^
Di2	(1/15.116) (1/544320)^1/8^	(1/12.616) (1/181440)^1/8^	(1/10.293) (1/45360)^1/8^
Di3	(1/15.116) (36)^1/8^	(1/12.616) (210)^1/8^	(1/10.293) (1120)^1/8^
Di4	(1/15.116) (1/30240)^1/8^	(1/12.616) (1/6720)^1/8^	(1/10.293) (1/1260)^1/8^
Di5	(1/15.116) (45360)^1/8^	(1/12.616) (181440)^1/8^	(1/10.293) (544320)^1/8^
Di6	(1/15.116) (1)^1/8^	(1/12.616) (6)^1/8^	(1/10.293) (35)^1/8^
Di7	(1/15.116) (1260)^1/8^	(1/12.616) (6720)^1/8^	(1/10.293) (30240)^1/8^
Di8	(1/15.116) (35)^1/8^	(1/12.616) (6)^1/8^	(1/10.293) (1)^1/8^

**Table 7:** Average and normal weightage.

Disorders	Mi	Ni
Di1	0.043	0.04115
Di2	0.018	0.01722
Di3	0.163	0.15598
Di4	0.028	0.02679
Di5	0.372	0.35598
Di6	0.105	0.10048
Di7	0.249	0.23828
Di8	0.067	0.06412

**Table 8:** Subcriteria priority computation.

Criteria	Subcriteria	Priority	Rank
Afflictive disorders	Lower back pain	0.04115	6
	Neck pain	0.01722	8

Specific disorders	Eyestrain	0.15598	3
	Hearing loss	0.02679	7

Psychosocial disorders	Mental stress	0.35598	1
	Fatigue	0.10048	4

Chronic disorders	Hypertension	0.23828	2
	Diabetes	0.06412	5

**Table 9:** Criteria priority computation.

Criteria	Priority	Rank
Afflictive disorders	0.05837	IV
Specific disorders	0.18277	III
Psychosocial disorders	0.45646	I
Chronic disorders	0.30240	II

**Table 10:** Comparison of our work with some recent contributions.

S. No.	Paper	Outcome	Present work
1	Daneshmandi et al. [67]	The authors observed that sedentary sitting is linked to MSD. They concluded that prolonged sitting behavior had adverse effects and suggested active workstations for improving the working environment.	The ergonomics-related disorders in online education such as lower back pain, neck pain, eyestrain, hearing loss, mental strain, fatigue, hypertension, and diabetes have been evaluated and compared.
2	Golabchi et al. [68]	The authors presented a fuzzy-based methodology for ergonomics evaluation by incorporating perceived differences in distinguishing human poses in the analysis system of ergonomics tactics.	Total eight ergonomics-related disorders (in categories such as afflictive disorders, specific disorders, psychosocial disorders, and chronic disorders) are compared using fuzzy analytical hierarchical process methodology to get ranked in terms of priority.
3	Metin and Yuksel [69]	The authors developed a model on the basis of calculating the best influencing parameters causing malfunctioning and applying precautionary measures in the correction of the parameters, using the fuzzy AHP method.	The present research work has demonstrated an MCDM modeling and assessment of total of eight ergonomic disorders (in four categories) in online education applying the fuzzy AHP technique.

## Data Availability

The data used to support the findings of this study are available from the corresponding author upon request.
